# Monitoring of the Physicochemical Properties and Aflatoxin of *Aspergillus flavus*-Contaminated Peanut Kernels Based on Near-Infrared Spectroscopy Combined with Machine Learning

**DOI:** 10.3390/foods14132186

**Published:** 2025-06-22

**Authors:** Yingge Wang, Mengke Li, Li Xu, Chun Gao, Cheng Wang, Lu Xu, Shaotong Jiang, Lili Cao, Min Pang

**Affiliations:** 1 School of Food and Bioengineering, Hefei University of Technology, Hefei 230009, China; wangyg0111@163.com (Y.W.); 18226129391@163.com (M.L.); 18747174360@163.com (L.X.); jiangshaotong@163.com (S.J.); lilycao504@hfut.edu.cn (L.C.); 2 Anhui Jiexun Optoelectronic Technology Co., Ltd., Hefei 230000, China; xuli2568@126.com (L.X.); gaochun1162@126.com (C.G.); wc5442wc@126.com (C.W.); 3 Key Laboratory for Agricultural Products Processing of Anhui Province, Hefei 230009, China

**Keywords:** aflatoxin, near-infrared spectrum, machine learning, correlation analysis, non-destructive technologies

## Abstract

This study explores the application of near-infrared (NIR) spectroscopy combined with machine learning for the non-destructive detection of aflatoxin in peanuts contaminated by *Aspergillus flavus* (*A. flavus*). The key innovation lies in the development of an optimized spectral processing pipeline that effectively overcomes moisture interference while maintaining high sensitivity to low aflatoxin concentrations. NIR spectra were collected from peanut samples at different incubation times within the spectral range of 950 to 1650 nm. Spectral data were preprocessed, and Competitive Adaptive Reweighted Sampling (CARS) selected ten characteristic bands. Correlation analysis was performed to examine the relationships between physicochemical properties, characteristic bands, and aflatoxin content. Three machine learning models—Backpropagation Neural Network (BPNN), Support Vector Machine (SVM), and Random Forest (RF)—were used to predict aflatoxin levels. The SNV-SVM model demonstrated superior performance, achieving calibration metrics (R^2^_C_ = 0.9945, RMSE_C_ = 9.92, RPD_C_ = 14.59) and prediction metrics (R^2^_P_ = 0.9528, RMSE_P_ = 19.58, RPD_P_ = 7.01), along with leave-one-out cross-validation (LOOCV) results (R^2^ = 0.9834, RMSE = 11.20). The results demonstrate that NIR spectroscopy combined with machine learning offers a rapid, non-destructive approach for aflatoxin detection in peanuts, with significant implications for food safety and agricultural quality control.

## 1. Introduction

Peanut is an annual herbaceous plant and a widely cultivated oilseed crop with significant economic value [1]. Peanut kernels are highly nutritious, containing up to 50% fat and 20% protein [2]. Notably, peanuts are rich in unsaturated fatty acids, which, when consumed in moderation, can help supplement essential fatty acids and potentially prevent or alleviate cardiovascular diseases. However, the high nutrient content of peanuts also makes them susceptible to mold growth and subsequent aflatoxin contamination during cultivation and storage.

Aflatoxin (AF) is a highly toxic and carcinogenic secondary metabolite produced by *Aspergillus flavus* and other parasitic fungi [3,4]. It is commonly found in soil and various cereals, with peanuts and wheat being particularly susceptible to contamination. The primary producer of AF is *A. flavus*, which synthesizes AF under favorable environmental conditions, such as temperatures of 28–32 °C and relative humidity above 80% [5]. Among the dozen known types of AF, Aflatoxin B_1_ (AFB_1_) is the most prevalent and poses significant health risks due to its potent carcinogenicity and toxicity. AFB_1_ contamination can occur in a wide range of food products. Acute exposure to high levels of AFB_1_ primarily damages the liver, leading to acute hepatitis and liver cell lesions, while chronic exposure can result in long-term liver damage and other health complications [6]. According to the Food and Agriculture Organization (FAO), approximately 25% of global agricultural products are contaminated with mycotoxins annually [7]. Given the susceptibility of peanuts to mold contamination during growth, storage, and transportation, the development of rapid, non-destructive methods for AF detection has become a critical area of research.

Various methods have been developed for the detection of aflatoxins (AF). Thin-layer chromatography (TLC) was the earliest method for AF detection [8], followed by other techniques such as gas chromatography–mass spectrometry (GC-MS) [9], high-performance liquid chromatography (HPLC) [10], and enzyme-linked immunosorbent assay (ELISA) [11]. However, these methods are often instrument-intensive, costly, and impractical for large-scale screening. In contrast, near-infrared (NIR) spectroscopy coupled with machine learning has emerged as a rapid, non-destructive, and cost-effective alternative for AF detection. NIR spectroscopy relies on molecular vibrational overtones and combination bands (mainly C-H, O-H, and N-H bonds), providing rich structural and compositional data [12]. This capability allows for the characterization of complex chemical compositions, including nutrient quantification (e.g., fats, proteins, starch) in agricultural products. NIR spectroscopy has shown great potential in grain mycotoxin detection.

The high dimensionality and large volume of NIR spectroscopy data necessitate the use of efficient data processing algorithms. Machine learning techniques can extract complex non-linear patterns from NIR spectral data, facilitating the development of accurate predictive models for AF content. These models automate the detection process, reduce human error, and facilitate intelligent data analysis, making them suitable for high-throughput detection of large-scale samples. For example, Bailly et al. [13] applied NIR spectroscopy combined with PCA-DA to classify cereal samples by AF contamination level, successfully distinguishing naturally contaminated maize. Similarly, Li et al. [14] developed a BWO-IVSO-SVM-based NIR model to predict AFB_1_ in peanuts, further validating the efficacy of NIRS coupled with chemometrics for mycotoxin analysis. Additionally, Carames et al. [15] demonstrated that an NIR-based PLS-DA model effectively discriminated contaminated from uncontaminated barley, reinforcing NIRS’s potential for mycotoxin prediction.

These studies demonstrate the potential of NIR spectra for aflatoxin detection in peanuts. However, existing research lacks real-time monitoring of physicochemical degradation and mycotoxin accumulation under varying environmental conditions over time. This study investigates the characteristic spectral features (950–1650 nm) of mold-damaged peanuts by incorporating temporal variables. This approach quantifies the relationship between mycotoxin accumulation and environmental factors. We analyze correlations between quality degradation indicators, aflatoxin concentrations, and key spectral bands to develop a robust mold detection model. By tracking dynamic spectral changes and mycotoxin production under controlled storage, we reveal time-dependent spectral responses linked to aflatoxigenic fungal growth. This approach surpasses static methods by capturing progressive biochemical changes during spoilage, improving early mold identification and risk prediction in stored peanuts.

## 2. Materials and Methods

### 2.1. Materials

*Aspergillus flavus* 2219 was purchased from the China Industrial Microbial Strain Preservation and Management Centre. The peanut variety used in the experiment was Silihong, purchased from the local supermarket. The AFB_1_ standard substance was purchased from Yifang Technology Co., Ltd. (Beijing, China), with a purity over 99% (HPLC). Acetonitrile and methanol were obtained from Macklin with a purity over 98% (Macklin Biotech Co, Ltd., Shanghai, China). All chemicals and organic solvents were of analytical grade and used as supplied. UltiMate 3000 High-Performance Liquid Chromatography was purchased from Thermo Fisher Scientific Co., Ltd. (Waltham, MA, USA), SE206 Fat Analyzer was purchased from ALRVA Co., Ltd. (Tokyo, Japan), K9840 Kjeldahl Nitrogen Analyzer was purchased from Hanon Group Co., Ltd. (Jinan, China)

### 2.2. Sample Cultivation

*A. flavus* was inoculated onto Wort Agar medium and cultured for 7 days. The spores were rinsed with sterile water to make the spore suspension, and the concentration of the spore suspension was adjusted to 1 × 10^5^ CFU/mL by the plate counting method before use. Twenty-four portions of 40 g peanuts were weighed using an electronic balance, and the spore suspension was sprayed evenly on the surface of the samples; the incubation temperatures were set at 28 °C and 15 °C, the moisture activity was set at three water activity(aw) conditions: ① 0.85 aw, ② 0.90 aw, and ③ 0.95 aw, and the number of days of incubation was set at 0, 3, 6, and 9 days. Random samples were taken from the samples at regular intervals for testing.

### 2.3. HPLC Analysis of Aflatoxins

In this study, the concentration of AFB_1_ in the samples was determined by high-performance liquid chromatography (HPLC).

#### 2.3.1. Standard Solution Preparation

Dissolve 1 mg of aflatoxin standard with acetonitrile to 100 mL, and the concentration of the solution is 10 μg/mL. Then, 10 μg/mL of standard solution will be diluted into a series of 1, 2, 5, 10, 20, 50, and 100 ng/mL standard solutions.

#### 2.3.2. Sample Preparation

The sample was pulverized and passed through a sieve with a particle size less than 2 mm. Weigh 5 g of sample in a 50 mL centrifuge tube, add 25 mL AFB_1_ extraction solution (Acetonitrile/Ultrapure water: 84 v/16 v), room temperature 200 r/min shaking extraction for 60 min, filtration of 10 mL filtrate, add 10 mL of trichloromethane extraction, vortex mixing for 1 min, stratification of the lower layer of the extraction solution, nitrogen blow-drying at 50 °C, add 200 μL acetonitrile aqueous solution of re-evaporated, add 700 μL of AFB_1_ derivatization solution (Trifluoroacetic acid/Ultrapure water/Glacial acetic acid: 20 v/70 v/10 v) and mix. Then, add 700 μL of AFB_1_ derivatization solution and mix well, and then add water at 40 °C for 75 min, vortex and mix well, and then pass through a 0.22 μm filter membrane. Each sample was analyzed with 3 independent HPLC measurements. Each analytical batch included standard calibration curves (R^2^ > 0.99).

#### 2.3.3. Chromatographic Conditions

Chromatographic column: Venusil MP C18(2) (4.6 mm × 250 mm, 5 μm); mobile phase A: water; mobile phase B: acetonitrile/methanol solution (50 v/50 v), gradient elution (0~6 min, 24% B; 8~12 min, 40% B; 13~17 min, 60% B; 18~22 min, 80% B; 23~27. The gradient elution (0–6 min, 24% B; 8–12 min, 40% B; 13–17 min, 60% B; 18–22 min, 80% B; 23–27 min, 100% B; 28–30 min, 24% B) was carried out at a flow rate of 1.0 mL/min, with a column temperature of 40 °C, detection wavelengths of 360 nm and 440 nm, and an injection volume of 10 μL.

### 2.4. Determination of Physicochemical Properties

The fat content was determined using the Soxhlet extraction method [16] outlined in GB 5009.6-2016 “Determination of Fat in Foods.” The acid value was measured using the petroleum ether extraction method [17] described in GB/T 5510-2011 “Inspection of Grain and Oil: Determination of Fatty Acid Value in Grains and Oilseeds.” Protein content was determined using the Kjeldahl method [18] as specified in GB 5009.5-2016 “Determination of Protein in Foods.” Starch content was measured using a plant starch content assay kit. Moisture content was determined by the direct drying method [19] in accordance with GB 5009.3-2016 “Determination of Moisture in Foods.” Each sample is detected three times, and the average value is taken.

### 2.5. NIR Spectroscopy Analysis

The spectra were collected at room temperature ((25 ± 2) °C), and the near-infrared (NIR) spectroscopy were collected using an Ocean Optics Flame-NIR (950–1650 nm) (Ocean Optic Instruments Co., Ltd. (Shanghai, China)) instrument, which was preheated for 30 min before the application of the spectrometer and corrected for black and white, and the NIR spectroscopy acquisition system of peanut samples was used in the fiber-optic diffuse reflectance mode for spectral acquisition (Figure S1). The spectra of each sample represented the average of three scans obtained from different points on the sample, and then the average value of each sample was calculated (Bruker OPUS 8.7).

### 2.6. Spectra Data Preprocessing

The raw spectral data were preprocessed as needed. The preprocessing of spectral data was all carried out in MATLAB R2023b. After preprocessing, the raw spectral data become more consistent in the whole, eliminating some of the noise, and the preprocessed spectral data are more suitable for subsequent analysis and modeling [20]. The spectral preprocessing method applied in this study:

1. Multiplicative Scatter Correction (MSC) is a method of spectral data preprocessing that removes spectral baseline drift caused by scattering effects and particle size differences and corrects the spectrum to a reference spectrum.

The formula for MSC is as follows:

① Calculation of spectral averages as reference spectra

(1) $$X_{ref}=\frac{1}{n}\sum_{i=1}^{n}X_{I}$$

② Linear regression for each measured spectrum

(2) $$X_{i}=a_{i}\cdot X_{ref}+b_{i}$$

③ Calculate the corrected spectrum

(3) $$X_{i}^{MSC}=\frac{X_{i}-b_{i}}{a_{i}}$$

2. Standard Normal Variate (SNV) is a data preprocessing method usually used for spectral data or other multivariate data. It aims to eliminate differences in measurements in the data and highlight systematic differences between samples, thereby improving the accuracy and reliability of modeling.

The formula for SNV is as follows:

(4) $$X_{i.SNV}=\frac{X_{i,K}-X_{i}}{\sqrt{\frac{\sum_{k=i}^{m}{\left(X_{i,k}-X_{i}\right)}^{2}}{\left(m-1\right)}}}$$

where X_i_ is the average of the spectra of the ith sample, k = 1, 2, ……, m. m is the number of wavelength points; i = 1, 2, ……, n. n is the number of calibration samples; X_i, SNV_ is the transformed spectrum.

3. Savitzky–Golay smoothing (SG smoothing) is a smoothing method commonly used in signal processing. It achieves smoothing by fitting a polynomial to the local data, and is particularly useful for removing noise and preserving the characteristics of the signal [21]. In this study, there is a window of 100 points and a polynomial order of 2.

### 2.7. Characteristic Bands Selection

Competitive Adaptive Re-weighting Strategy (CARS) is an algorithm for machine learning and optimization problems. Its core idea is to optimize the performance of a model or strategy by dynamically adjusting the weights [22]. This study collected spectral data from peanut samples spanning 128 wavelength bands between 950 and 1650 nm, with preprocessing steps applied to remove moisture and baseline interference effects. The Competitive Adaptive Reweighted Sampling (CARS) algorithm was implemented with 50 sampling iterations and 5-fold cross-validation, utilizing 2 PLS latent variables while maintaining an exponential decay rate (α) of 0.92 and an initial variable retention ratio of 90%. The analytical procedure began with the full wavelength spectrum as the initial variable set, where a PLS regression model was first constructed to obtain absolute regression coefficients (|β|) for each wavelength. During iterative processing, the number of retained variables followed a progressively decreasing trend, with wavelength selection prioritized according to |β| magnitudes to preserve spectrally significant features. Monte Carlo sampling was incorporated at each iteration through random subsampling to recalculate coefficient weights, thereby avoiding local optima while enhancing model robustness. The optimal wavelength subset was determined by evaluating the root mean square error of cross-validation (RMSECV) at each stage, with the final selection corresponding to the spectral features yielding minimal prediction error. This systematic integration of coefficient-weighted variable ranking, stochastic resampling, and cross-validated error minimization ensured both analytical precision in wavelength selection and model reliability for subsequent aflatoxin quantification. The purpose of CARS is to improve the model’s adaptability and performance by dynamically adjusting the weights, which is particularly suitable for dealing with complex, multi-objective optimization problems [23].

### 2.8. Correlation Analysis

Pearson correlation analysis is a statistical technique employed to assess the strength and direction of a linear relationship between two continuous variables. In this study, IBM SPSS Statistic 27 was utilized to analyze the physical and chemical properties, toxin content, and spectral bands of the samples. The analysis involved calculating the Pearson correlation coefficient (denoted as r) between pairs of variables. The coefficient r ranges from −1 to 1, where r = 1: indicates a perfect positive linear correlation, meaning that as variable X increases, variable Y increases proportionally; r = −1: indicates a perfect negative linear correlation, meaning that as variable X increases, variable Y decreases proportionally; r = 0: suggests no linear relationship between the variables, although other types of relationships (e.g., nonlinear) may exist; −1 < r < 1: the closer the absolute value of r is to 1, the stronger the linear relationship between variables X and Y. Specifically, values closer to 1 indicate a stronger positive linear relationship, while values closer to −1 indicate a stronger negative linear relationship. The results of the Pearson correlation analysis were visualized using a correlation heat map generated in MATLAB R2023b, providing a clear and intuitive representation of the relationships among the variables.

### 2.9. Machine Learning Arithmetic

The characteristic bands were modeled, and the prediction models used in this study are as follows:

1. Backpropagation Neural Network (BPNN) is a method of prediction using neural networks, which learns complex nonlinear mapping relationships between inputs and outputs by training neural networks. Its operational steps are coupled with dividing the data into training, validation, and test sets, then determining the architecture of the neural network, including the number of layers, the number of neurons per layer, and the activation function, etc., performing forward propagation and backpropagation through the training set, adjusting the weights to minimize the loss function, evaluating the performance of the model using the validation set, adjusting the parameters of the model to improve the accuracy, and finally, performing the prediction on the test set and evaluating the model’s generalization ability [24]. The parameters of the model are shown in Table S1.
2. Support Vector Machine (SVM) is a powerful supervised learning algorithm that can be used for regression prediction. SVM operates by finding hyperplanes in high-dimensional feature space, effectively separating data by categories and maximizing the intervals between the categories, which in turn improves the robustness and accuracy of the classification [25]. SVM is popular and is known to be powerful in classifying and handling high-dimensional data, and the implementation of kernel functions and penalty parameters enables the algorithm to handle classification tasks that contain linear and nonlinear distinctions [26], but there are some challenges in handling large-scale data, model tuning, and interpretability. The parameters of the model are shown in Table S2.
3. Random Forest (RF) is an integrated learning method that improves the predictive power and stability of a model by combining multiple decision trees. Random Forest improves prediction accuracy by constructing multiple decision trees and combining their predictions. Each tree is trained on a different subset of training data, and the predictions are determined by classification or regression [27]. Random forests are widely used for tasks such as classification, regression, feature selection, etc., and are a powerful and flexible machine learning method. The parameters of this model are shown in Table S3.

### 2.10. Model Evaluation

Coefficient of Determination (R^2^), Root Mean Square Error (RMSE), and Residual Prediction Deviation (RPD) are used as regression model evaluation indices in this study. To avoid model over-fitting in evaluating the effectiveness of multivariate statistical models, leave-one-out cross-validation (LOOCV) is used.

1. R^2^ indicates the proportion of the model that accounts for the data variation; the formula for R^2^ is as follows:

(5) $$R^{2}=1-\frac{\sum_{i=1}^{n}{\left(y_{i}-{\hat{y}}_{i}\right)}^{2}}{\sum_{i=1}^{n}{\left(y_{i}-{\bar{y}}_{i}\right)}^{2}}$$

$y_{i}$ is the ith true value, ${\hat{y}}_{i}$ is the ith predicted value, ${\bar{y}}_{i}$ is the mean of the true values: $\bar{y}=\frac{1}{n}\sum_{i=1}^{n}y_{i}$. n is the number of samples.

2. RMSE is the square root of MSE, which measures the standard deviation between predicted and actual values to establish a stable prediction model; the formula for RMSE is as follows:

(6) $$RMSE=\sqrt{\frac{1}{n}\sum_{i=1}^{n}{\left(y_{i}-{\hat{y}}_{i}\right)}^{2}}$$

$y_{i}$ is the ith true value, ${\hat{y}}_{i}$ is the ith predicted value, n is the number of samples.

3. RPD is a metric used to evaluate the predictive performance of regression models; the formula for RPD is as follows:

(7) $$RPD=\frac{SD}{RMSE}$$

SD is Standard Deviation of reference values, and RMSE is Root Mean Square Error.

## 3. Results

### 3.1. Occurrence of AFB_1_ in Peanut Samples by HPLC

**Figure 1:**
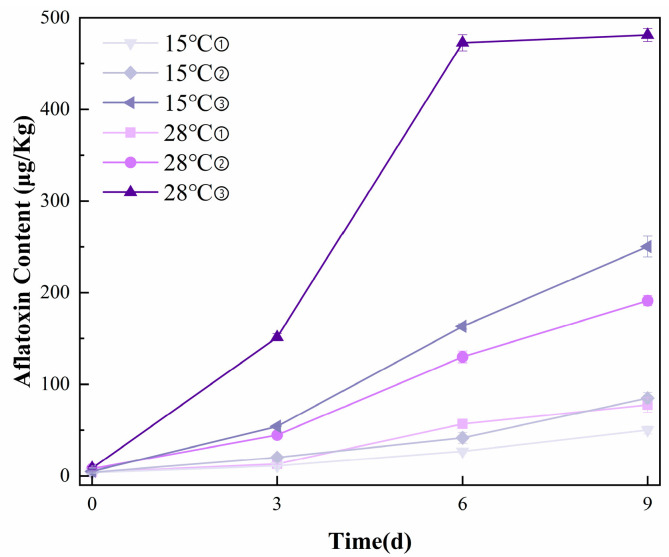
Reference measurement results of AFB_1_ content in peanuts with HPLC. (① 0.85aw, ② 0.90aw, ③ 0.95aw).

Figure 1 presents the HPLC analysis results of peanut samples. The retention time of the aflatoxin standard and sample is approximately 13.187 min, and aflatoxin concentrations ranged from 3.12 to 488.64 μg/kg across samples. Overall, aflatoxin levels exhibited a continuous accumulation trend with increasing incubation time. Notably, the peanut samples incubated for 9 days exhibited the highest average AFB_1_ content of 481.34 μg/kg, significantly exceeding the Chinese national food safety standard limit of 20 μg/kg for AFB_1_ in peanuts. In the later stages of incubation, the rate of aflatoxin accumulation slowed, which may be attributed to limited space and nutrient availability, leading to the inhibition or death of some fungal populations. This observation suggests that environmental constraints during incubation can influence fungal activity and, consequently, aflatoxin production.

### 3.2. Physicochemical Property Changes in Peanut Samples

**Figure 2:**
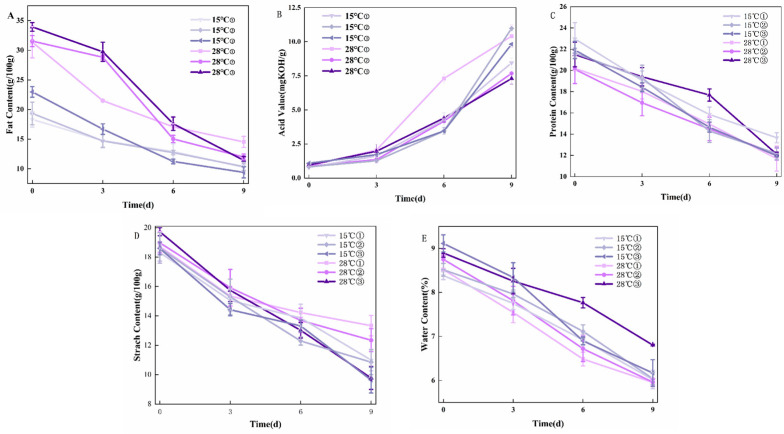
Changes in physicochemical properties of moldy peanuts during storage: (**A**) Fat content; (**B**) Acid value; (**C**) Protein content; (**D**) Starch content; and (**E**) Water content. (① 0.85aw, ② 0.90aw, ③ 0.95aw).

The fat content of the samples showed a consistent decline, as illustrated in Figure 2A. The decline was more pronounced from 0 to 6 days of incubation, with mean reductions of 20 g/100 g in the fat content of peanut samples incubated at 28 °C, and 9 g/100 g in those incubated at 15 °C. The observed fat loss likely resulted from the metabolic activity of mold, which relies on extracellular enzymes to hydrolyze macromolecules such as fats and proteins into soluble low-molecular-weight compounds [28]. These compounds are then absorbed and utilized by fungal cells for growth and energy. Notably, *A. flavus* secretes abundant enzymes during its growth, especially under high-humidity conditions. These enzymes facilitate the degradation of fats and other organic components in peanuts, leading to the observed decrease in fat content. Such enzymatic degradation is central to fungal metabolism and contributes to the overall changes in the nutritional composition of peanuts during incubation.

The acid value of peanut samples showed a strong positive correlation with incubation duration, as illustrated in Figure 2B. Specifically, the acid value of peanut samples incubated at 28 °C with a humidity of 0.85 aw sharply increased from 2.05 mg KOH/g to 7.31 mg KOH/g during days 3–6 of incubation. Similarly, for samples incubated at 15 °C with a humidity of 0.90 aw, the acid value rose markedly from 3.45 mg KOH/g to 10.98 mg KOH/g during the 6–9-day incubation period. This acid value elevation primarily resulted from the growth and proliferation of molds during the incubation process. As molds develop, they secrete enzymes that hydrolyze fats, leading to the production of free fatty acids and other acidic compounds. Consequently, the acid value of peanuts demonstrates a consistent upward trend over time. Notably, linoleic acid (a dominant peanut fatty acid) accumulates during storage [29], further elevating acid values.

As shown in Figure 2C., after 9 days of incubation, the samples incubated at 15 °C with a humidity of 0.90 aw exhibited the highest protein content, measuring 12.18 g/100 g. Over the 0–9-day incubation period, the average decrease in protein content for peanut samples incubated at 28 °C was 8.62 g/100 g, while for those incubated at 15 °C, the average decrease was 9.57 g/100 g. These results indicate that both temperature and humidity significantly influence the degradation of protein content in peanut samples during incubation. The growth and metabolism of molds in the process of peanut molding also consume protein and other nutrients [30]. *A. flavus* produces aflatoxins during its growth, and these toxins may further affect the protein synthesis and catabolism processes in peanuts, leading to accelerated protein degradation.

As observed in Figure 2D, after 9 days of incubation, the samples incubated at 28 °C with a humidity of 0.85 aw showed the highest starch content, reaching 13.33 g/100 g, whereas the samples incubated at 15 °C with a humidity of 0.95 aw had the lowest starch content, at 9.63 g/100 g. These findings highlight the significant influence of temperature and humidity on the starch content of the samples during the incubation period. During the process of peanut molding, carbohydrates such as starch will be hydrolyzed, and the metabolism of molds will consume carbohydrates as the molds continue to grow, so the starch content of peanut samples will be different in different culture conditions.

When the incubation days were 9 days, the moisture content of the samples with humidity of 0.95 aw at 28 °C was the highest at 6.80 g/100 g (Figure 2E). From 0 to 9 days, the mean value of the decrease in the moisture content of peanut samples incubated under the condition of 28 °C was 2.48 g/100 g, and the mean value of the decrease in the moisture content of the samples incubated at 15 °C was 2.59 g/100 g. The moisture in the peanuts would be affected by temperature and humidity, which would then affect the intrinsic quality of peanuts. Moisture in peanuts is affected by temperature and humidity, which in turn affects the intrinsic quality of peanuts. Most varieties of peanuts have a fat content between 46% and 54% and a protein content between 24% and 36% [31], and in addition to fat and protein, they also contain 10% to 23% of carbohydrates, of which proteins and starch, among others, are highly hydrophilic. Therefore, changes in the external environment are likely to cause changes in the moisture content of peanuts.

### 3.3. Analysis of Spectral Data Preprocessing Results

**Figure 3:**
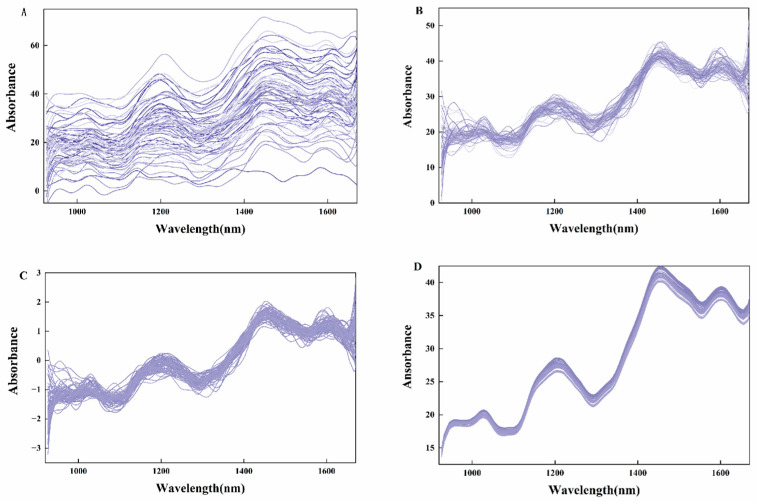
(**A**) Original spectrum of moldy peanut; (**B**) Spectrum pretreated with MSC; (**C**) Spectrum pretreated with SNV; and (**D**) Spectrum pretreated with SG.

Spectral preprocessing is critical for ensuring accurate, reliable, and analyzable spectral data. Three preprocessing methods—MSC, SNV, and Savitzky–Golay (SG) smoothing—were applied to the spectral data (Figure 3). MSC corrects baseline drift to reduce the effect of baseline drift on the spectra and removes spectral signal variations caused by sample scattering, making the spectral data more relevant to the chemical composition of the sample. SNV centers spectra to zero-mean and scales to unit variance by subtracting the mean and dividing by the standard deviation, which removes the spectral amplitude variations caused by the sample’s physical state. This eliminates variations in spectral amplitude caused by the physical state of the sample. SG smoothing enhances spectral smoothness and sharpens peak/trough resolution, facilitating subsequent qualitative and quantitative analysis.

The preprocessed spectral data are better suited for building subsequent regression prediction models. Competitive adaptive reweighted sampling is a variable selection method that combines Monte Carlo sampling with regression coefficients for PLS models to mimic natural selection principles for spectral band optimization [32], iteratively eliminating irrelevant bands while retaining the most predictive features.

**Figure 4:**
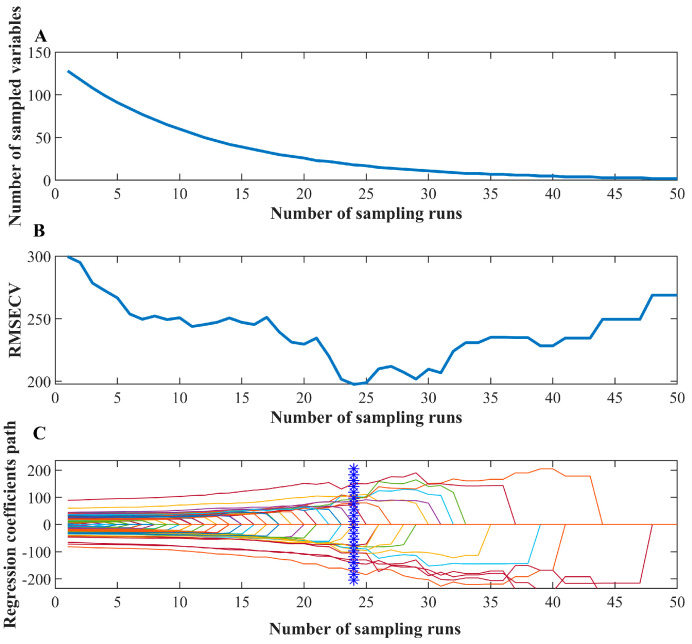
CARS variable selection results: (**A**) Variation trend of acquired variables number with the iteration; (**B**) RMSECV value variation with the iteration, and (**C**) Regression coefficient variation with the iteration (The blue mark represents the optimal number of iterations).

**Figure 5:**
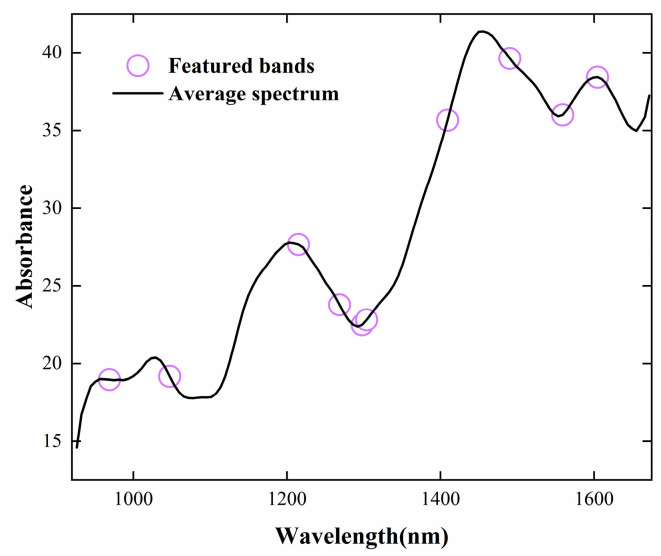
Featured bands of CARS selection for quantitative modeling of aflatoxin content.

CARS was applied to select informative spectral features of NIR data with peanut samples. The process of variable screening is shown in Figure 4. It can be seen that the optimal number of iterations is 24, and the band yielding the lowest RMSECV was selected as the characteristic band [33]. The characteristic bands marked in Figure 5 are 968.798, 1047.373, 1215.063, 1268.433, 1297.958, 1303.851, 1409.258, 1490.279, 1558.988, and 1604.39 nm. The average response rate of moldy peanuts in the wavelength range of 950–1650 nm was lower than that of healthy peanuts, with obvious spectral response characteristics. The band near 968 nm was mainly related to the second-order overtones of the O-H stretching state of water; the band near 1215 nm was related to the second-order overtones of the C-H bonding of methyl groups, and the deformation modes differed significantly between low- and high-aflatoxin groups; the band near 1270 nm corresponds to C-C stretching and C-H bending vibrations. The band at 1488 nm is related to the second-order overtones of the O-H stretching state of water, and these deformation modes correlated with aflatoxin contamination levels [34]. The near-infrared spectral region is consistent with the absorption region of the combined frequency and all levels of the octave of the vibration of hydrogen-containing groups in organic molecules [35]. The major NIR bands associated with aflatoxin molecules include 1050 (C–H bond stretch) and 1410 nm (C–H stretching and deformation) [36] and the bands near 1600 nm corresponds to the second overtone of C=O stretching vibration, which is associated with the lactone or carboxyl group structures in aflatoxin molecules. When peanuts undergo mold growth, their chemical composition changes, such as C-H and C-C groups, which are usually associated with the content of fats, proteins, volatile or non-volatile acids, alkaloids, etc. [37]. The level of aflatoxin increases with the increase in the number of days of incubation. During molding or sprouting, peanuts undergo nutrient decomposition due to mold growth and respiration, altering fat and protein contents, which affects the response values in the range of different bands.

### 3.4. Correlation Analysis

**Figure 6:**
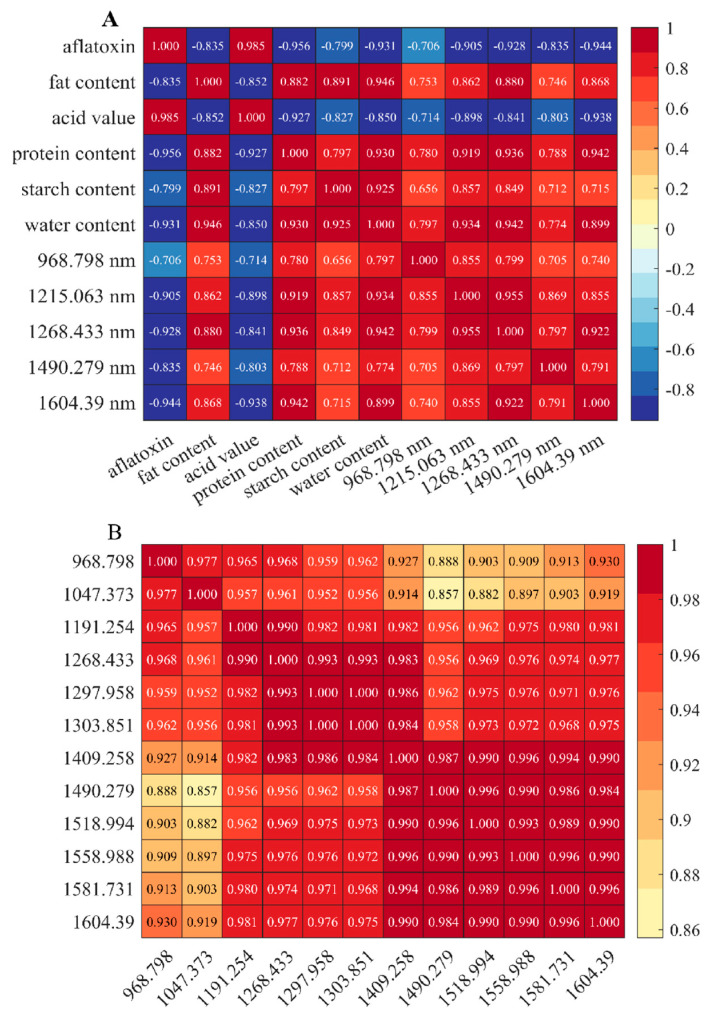
Correlation heat map: (**A**) Correlation heat map of quality, aflatoxin, and feature bands; (**B**) Correlation heat map of feature bands.

We used Pearson correlation analysis to measure the strength and direction of the linear relationship between variables [38]. It calculates the ratio of covariance between two variables to the product of their standard deviations. Pearson correlation analysis is computationally efficient, intuitive, and sensitive to linear relationships, and can quickly provide correlation coefficients to help understand the association between variables. As shown in Figure 6, aflatoxin content was strongly positively correlated with acid value (r = 0.985, *p* < 0.01) but negatively correlated with fat content, protein content, and other variables. The heatmap revealed significant associations among moldy peanuts’ intrinsic quality, toxin levels, and NIR characteristic bands. These features could serve as indicators for assessing peanut mold severity.

### 3.5. Analysis of Regression Prediction Model

**Figure 7:**
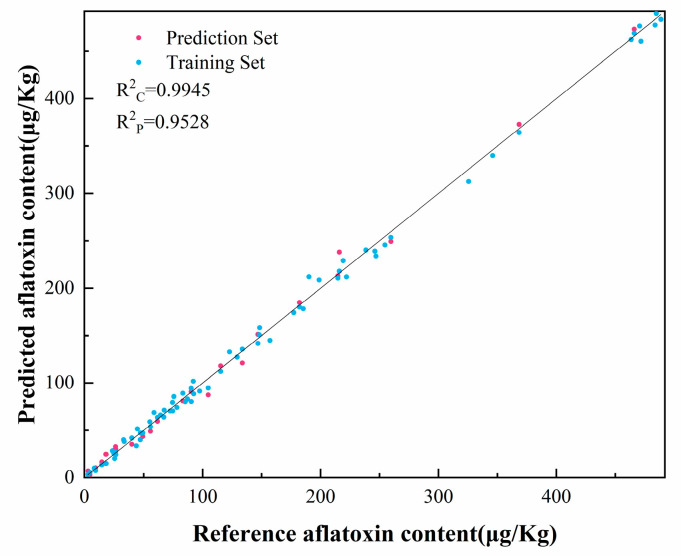
Regression prediction based on SVM of aflatoxin content of the spectral data pretreated with SNV.

For modeling moldy peanut contamination, a regression prediction model was established utilizing 72 samples. The dataset was strategically divided employing the Kennard–Stone (K-S) algorithm at a 7:3 ratio, comprising 50 samples for model calibration and 22 samples for prediction validation. We performed regression predictions using BPNN, SVM, and RF of the original spectra and the feature bands processed by different preprocessing methods, respectively, and the performance of different preprocessing methods and regression models was compared. From Table 1, it can be seen that preprocessed spectra yielded higher prediction accuracy than raw spectra, in which the regression prediction model built by SVM using SNV for preprocessing has the best prediction accuracy, and R^2^_C_ of this model is 0.9945, R^2^_P_ is 0.9528, RMSE_C_ is 9.92, RMSE_P_ is 19.58, RPD_C_ is 14.59, RPD_P_ is 7.01, LOOCV-R^2^ is 0.9834, and RMSE is 11.20. These data show that SVM can predict aflatoxin content of moldy peanuts better, and model generalizability and robustness may be further enhanced with larger sample sizes in subsequent studies. Figure 7 demonstrates the results of the regression prediction based on the SVM using SNV for preprocessing, and all the samples were distributed on both sides of the center line, which indicated that the reference AFB_1_ content confirmed a good linear relationship with the predicted values of the NIR spectra. These results demonstrate the method’s strong predictive capability for identifying *A. flavus*-contaminated peanuts.

## 4. Discussion

In this study, near-infrared (NIR) spectroscopy combined with machine learning was employed to achieve rapid and non-destructive detection of intrinsic quality deterioration in moldy peanuts. This study involved the incubation of peanut samples at 28 °C and 15 °C, with water activity levels set at 0.85 aw, 0.90 aw, and 0.95 aw, over a period of 0, 3, 6, and 9 days. The optimal growth temperature for Aspergillus flavus is 28 °C, which facilitates rapid proliferation and mycotoxin production, while 15 °C represents potential low-temperature conditions encountered during storage or transportation. The minimum water activity (aw) for A. flavus growth is approximately 0.82, with higher aw required for toxin production. An aw of 0.85 approaches the lower growth limit, enabling investigation of contamination risks under marginal conditions; 0.90 aw simulates moderately humid storage environments commonly observed in peanuts; and 0.95 aw significantly promotes fungal growth and toxin accumulation, encompassing scenarios from safe storage to high-risk conditions. The incubation period was dynamically monitored at 0, 3, 6, and 9 days to characterize contamination progression. Day 0 served as the blank control to establish baseline contamination levels. Day 3 captured initial growth phases and incipient toxin synthesis, while Day 6 represented the logarithmic growth phase for evaluating contamination during rapid proliferation. Day 9 corresponded to the stationary phase to observe maximum toxin accumulation. This temporal framework comprehensively covers typical exposure durations from short-term to extended storage periods.

The results demonstrated that NIR spectroscopy could serve as an effective alternative method for the efficient and rapid analysis of mold contamination. The aflatoxin content in the peanut samples, as quantified by high-performance liquid chromatography (HPLC), ranged from 3.12 to 803.68 μg/kg. During the molding process, the nutritional composition of the peanuts underwent significant changes: the fat and protein content decreased, while the acidity increased. These alterations were attributed to the metabolic activity of mold, which consumes nutrients such as fats and proteins to support its growth and proliferation.

NIR spectra of moldy peanuts were acquired, and the spectral data underwent preprocessing via multiplicative scatter correction (MSC), standard normal variate (SNV), and Savitzky–Golay (SG) smoothing to eliminate noise. Competitive adaptive reweighted sampling (CARS) was applied to select the characteristic bands with the minimal root mean square error of cross-validation (RMSECV) from the preprocessed spectra. Correlation analysis of aflatoxin content, physicochemical properties, and characteristic bands revealed that these physicochemical properties and characteristic bands could serve as effective indicators for assessing the degree of mold contamination in peanuts. The selected bands were modeled via BPNN, SVM, and RF algorithms, with subsequent performance comparison. The results demonstrated that the regression prediction model utilizing SNV for spectral data preprocessing combined with SVM exhibited the best predictive performance.

## 5. Conclusions

In summary, the established SNV-SVM model demonstrated feasibility, proving that the short-wave region (950–1650 nm) can effectively preserve the characteristic peaks of AFB_1_ while reducing moisture interference. The selected feature wavelengths can be directly applied to the development of multispectral imaging sorting equipment, thereby facilitating industrial applications. This study has achieved innovations in several aspects, including moisture interference reduction, optimized feature selection, and quantitative assessment of environmental factors, by integrating short-wave near-infrared spectroscopy with machine learning. According to literature reports [39], the characteristic wavelength points obtained in this study are basically consistent with the key wavelength points of aflatoxin B_1_ in the near-infrared spectral region. The proposed approach provides a reliable solution for rapid, non-destructive detection of aflatoxin in peanuts. However, it should be noted that the current method is primarily suitable for screening moderate to high contamination levels, while low-concentration samples still require confirmatory methods such as HPLC. Further validation using certified reference materials and naturally contaminated samples is needed to verify the method’s robustness, and the inclusion of non-toxigenic *A. flavus* strains would help confirm the toxin-specificity of spectral features. Despite these areas for improvement, the stepwise modeling strategy and feature wavelength screening method developed in this study lay an important theoretical foundation for portable detection device development, industrial standardization implementation, and extension to peanuts of different varieties and origins. Future research should focus on validation with broader samples under various storage conditions, optimization of low-concentration detection performance, and establishment of industrial application standards.

## Figures and Tables

**Table 1:** Performance of various regression models with different spectral preprocessing methods.

Prediction Models	Preprocessing	Calibration Set			Prediction Set			LOOCV	
		R^2^_C_	RMSE_C_	RPD_C_	R^2^_P_	RMSE_P_	RPD_P_	R^2^	RMSE
BPNN	Orig. spec	0.4554	96.49	1.49	0.1218	91.96	1.11	0.2451	93.16
	MSC	0.9176	37.85	3.82	0.3971	73.60	1.39	0.7523	55.35
	SNV	0.9589	28.50	5.08	0.5616	29.16	3.51	0.8143	28.73
	SG	0.6059	140.51	1.03	0.1664	357.96	0.29	0.4317	214.62
SVM	Orig. spec	0.3808	106.06	1.36	0.6050	50.66	2.02	0.5872	64.28
	MSC	0.9516	29.66	4.88	0.6740	49.10	2.08	0.8456	34.58
	**SNV**	**0.9945**	**9.92**	**14.59**	**0.9528**	**19.58**	**7.01**	**0.9834**	**11.20**
	SG	0.2185	298.15	0.49	0.3307	73.71	1.39	0.2562	102.34
RF	Orig. spec	0.4296	98.05	1.48	0.4144	50.66	2.02	0.4157	61.35
	MSC	0.5616	90.18	1.60	0.7097	41.83	2.44	0.7011	46.57
	SNV	0.5797	88.81	1.63	0.7446	37.91	2.70	0.6781	47.37
	SG	0.3920	211.38	0.68	0.2378	279.07	0.37	0.3914	223.45

BPNN—Backpropagation Neural Network, SVM—Support Vector Machine, RF—Random Forest, Orig. spec—original spectrum, MSC—Multiplicative Scatter Correction, SNV—Standard Normal Variate, SG—Savitzky–Golay smoothing, LOOCV—Leave-One-Out Cross-Validation. Bold indicates the optimal preprocessing methods.

## Data Availability

The data that support the findings of this study are available upon request by contacting the corresponding author.

## Supplementary Materials

The following supporting information can be downloaded at: https://www.mdpi.com/article/10.3390/foods14132186/s1, Figure S1: Schematic diagram of near-infrared spectral collection system; Table S1: Parameters of BPNN model; Table S2: Parameters of SVM model; Table S3: Parameters of RF model.
