# Supplementary material for: Monitoring of the Physicochemical Properties and Aflatoxin of Aspergillus flavus-Contaminated Peanut Kernels Based on Near-Infrared Spectroscopy Combined with Machine Learning
Source: Foods. 2025 Jun 22;14(13):2186. doi: 10.3390/foods14132186 (PMC12249350; doi:10.3390/foods14132186)
Supplement: Supplementary file 1 [file foods-14-02186-s001.zip › foods-3699444-supplementary.pdf]

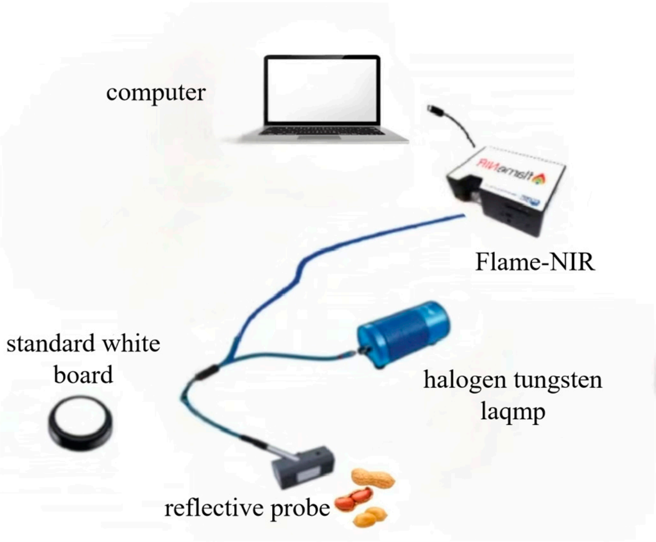


**Figure S1. Schematic diagram of near-infrared spectral collection system**

**Table S1. Parameters of BPNN model**

| model | calibration set samples | prediction set samples | hidden layers | maximum iteration | goal error | learning rate |
|---|---|---|---|---|---|---|
| BPNN | 50 | 22 | 5 | 1000 | 1e-6 | 0.01 |

*** BPNN Backpropagation Neural Network**

**Table S2. Parameters of SVM model**

| model | calibration set samples | prediction set samples | penalty factor | gamma | epsilon |
|---|---|---|---|---|---|
| SVM | 50 | 22 | 4.0 | 0.8 | 0.01 |

*** SVM Support Vector Machine**

**Table S3. Parameters of RF model**

| model | calibration set samples | prediction set samples | trees | leaf |
|---|---|---|---|---|
| RF | 50 | 22 | 100 | 5 |

*** RF Random Forest**
